# Fahr's Disease (Primary Basal Ganglia Calcification) and Violence: Case Report and Literature Review

**DOI:** 10.1192/bjo.2024.695

**Published:** 2024-08-01

**Authors:** Graeme Yorston, Sidney Mumonyedi, Muhammad Abdur Rehman, Ifrah Ali Baig

**Affiliations:** St Matthew's Healthcare, Northampton, United Kingdom

## Abstract

**Aims:**

Background: Fahr's disease is a rare and complex neuropsychiatric disorder resulting from abnormal calcium deposition in the basal ganglia and cerebral cortex. It can have a profound impact on an individual's social functioning as well as causing a wide variety of neurological symptoms, cognitive deficits and motor impairment. A number of specific mutations have recently been identified in phosphate transporter and other genes, but around half of all cases have unidentified mutations. Impulsivity, aggression and violence may pre-date the other manifestations of the illness.

**Methods:**

Case Report: Patient X is a 58 year old man currently detained in an independent hospital locked rehabilitation unit following the breakdown of a care home placement. His first admission to hospital was at the age of 18 when he was diagnosed with mania. He had multiple further hospital admissions as well criminal convictions for acquisitive and violent offences. In 2005 he threatened to stab a stranger if he did not give him a cigarette and he was arrested and admitted to a medium secure unit under Section 37 with diagnoses of bipolar affective disorder and emotionally unstable personality disorder. He remained in secure hospital care until 2018 when concerns about Parkinsonian symptoms led to him being referred to a neurologist and a diagnosis of Fahr's disease being made on the basis of his CT findings. He was transferred to a locked rehabilitation service in 2019 but continued to exhibit challenging behaviour on a daily basis. After a reduction in the frequency and severity of his behaviour he was discharged to a care home, but this broke down after a few months as his assaultive and sexually inappropriate behaviour re-emerged.

**Results:**

Discussion: Fahr's disease is traditionally thought of as a late life neurological condition, but as with Huntington's disease neuropsychiatric symptoms of irritability, sexually disinhibited behaviour, impulsivity and aggression can occur early and may pre-date any neurological manifestations. Treatment is often difficult because of sensitivity to antipsychotic medication.

**Conclusion:**

It is important to consider neuropsychiatric conditions in the assessment of adults presenting with antisocial behaviours, especially when these are associated with a change in overall functioning and an absence of adolescent conduct disorder. There is as yet no specific treatment for Fahr's disease, but early identification allows appropriate risk management strategies to be adopted.

